# Clinical data and risk factors for diabetic nephropathy in Brazilian central population

**DOI:** 10.1016/j.dib.2018.10.115

**Published:** 2018-10-27

**Authors:** Elisangela Gomes da Silva, Laura Raniere Borges dos Anjos, Rayane Mendes de Lima, Thales Biffe Alves, Gustavo Rodrigues Pedrino, Aline Helena da Silva Cruz, Rodrigo da Silva Santos, André Henrique Freiria-Oliveira, Angela Adamski da Silva Reis

**Affiliations:** a Laboratory of Molecular Pathology, Institute of Biological Sciences (ICB), Federal University of Goiás (UFG), Goiânia, GO, Brazil; b Biological Sciences Institute (ICB), Federal University of Goiás (UFG), Goiânia, GO, Brazil; c Department of Nature Sciences (LEdoC), Special Academic Unit of Human Sciences, Federal University of Goiás (UFG), Goiás- GO, Brazil; d Biological Sciences, Education and Culture Society of Goiás, Araguaia Faculty, Goiânia, GO, Brazil

## Abstract

This article describes data set of the profile of patients diagnosed with Diabetic Nephropathy (DN) undergoing hemodialysis and followed-up by Hemodialysis Service in medical centers in Goiânia, Go, Brazil. These data describe specifically the demographic, clinical, and lifestyle variables of 101 patients. In addition, these data provide detailed clinical associations about the profile of patients diagnosed with DN and which are made publicly available to enable critical or extended analyzes. For further interpretation of the data presented in this article, see the research article: Do GST polymorphisms influence in the pathogenesis of diabetic nephropathy? (Lima et al., 2018).

**Specifications table**

**Table:** 

Subject area	*Endocrinology*
More specific subject area	*Diabetic Nephropathy*
Type of data	*Table and figure*
How data was acquired	*The data were collected in medical centers in the metropolitan region of Goiânia, Go, Brazil. The data were processed in the RStudio Software v.1.0.153*
Data format	*Raw analysis*
Experimental factors	*The information on demographic features, lifestyle, time with type 2 Diabetes mellitus and the main exams associated with DN control were collected through questionnaires and clinical records’ analysis.*
Experimental features	*The parameters analyzed are according to the criteria established by the American Diabetes Association (ADA) as a reference for the data analysis.*
Data source location	*Central Brazil*
Data accessibility	All data are presented in this article
Related research article	*R.M. Lima, L.R.B. dos Anjos, T.B. Alves,A.S.G. Coelho, G.R. Pedrino, R.S. Santos, A.H.S. Cruz, A.A.S. Reis. Do GST polymorphisms influence in the pathogenesis of diabetic nephropathy? Mol Cell Endocrinol. 478 (2018) 10–16*[1].

**Value of the data**

• The data show the hyperglycemia may negatively influence the diabetes mellitus (DM) patient׳s clinical status for diabetic nephropathy (DN) development.
• The pre-hemodialysis patients’ presented high-level blood urea, due to the presence of an inadequate diet and /or inadequate treatment, respectively. However, the increased in the level of urea was not consistently associated with the reduction of GFR.
• The dataset demonstrated that the smoking habits contributed to DN development in association with others risk factors.
• These data may be relevant, due to the prevalence of 76.24% of the patients with blood pressure levels inconsistent, indicating systemic arterial hypertension associated with DM act as comorbidity factors for DN development.
• These data allow other researchers to extend the statistical analyses.

## 1 Data

**Fig. 1:**
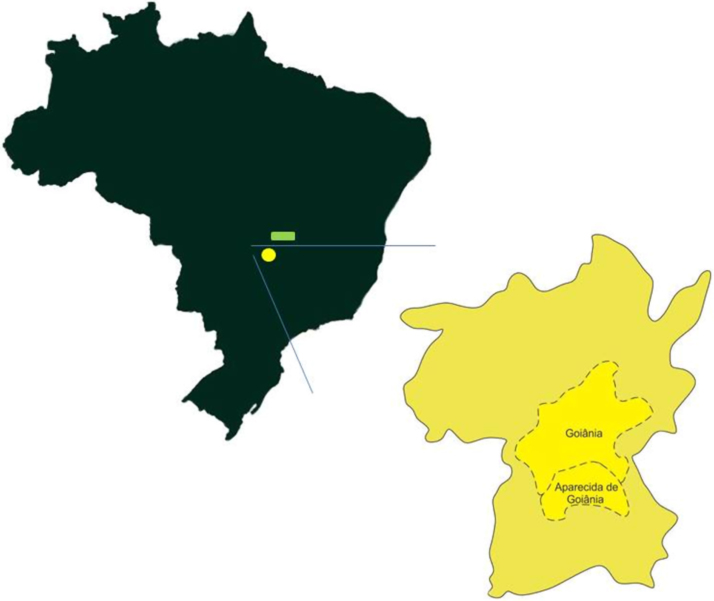
Metropolitan region of Goiânia, Aparecida de Goiânia , GO, Brazil.

The demographic and clinical variables features of the studied population are described in Tables 1, 2 and 3. Clinical features of the patients with DN are described in Table 4. Blood pressure of the patients with DN is described in Table 5. In addition, it was detected that 18.81% and 28.71% of the patients presented obese and overweight, respectively (Table 6). For proper dietary enrollment, 27.72% of the patients follow an adequate diet, and 72.28% have issues in following an appropriate diet due to financial issues and difficulty (Table 7). When analyzing clinical variables of patients who did or did not follow an adequate diet, a significant difference between these groups was observed in some aspects. The creatinine ratio (mean 7.46, *p* = 0.04), GFR (10.23, *p* < 0.001) and DAP (mean 81.43, *p* = 0.006) were higher in subjects who did not follow the diet correctly (Table 8). Fig. 1 describes the Metropolitan region of Goiânia, Aparecida de Goiânia, GO, Brazil.

**Table 1:** Demographic features of the patients with DN.

**Variable**	**Male**	**Female**		***p*-Valor**		**Total**	
*n*, %	58	57.43	43	42.53	–	101	100%
Age (years), $\bar{X}$ and ±	59.36	11.19	62.19	12.48	0.24	60.56	11.78
DM2 involvement time (years), $\bar{X}$ and ±	15.28	10.22	18.38	9.22	0.13	16.67	9.86

The data are shown as averages ($\bar{X}$), standard deviation (±) and frequency absolute and relative. *p* < 0.05 = level of significance.

**Table 2:** Distribution of the patients with DN based on their age range for gender.

**Age range (years)**	**DN in group female**		**DN in group male**		**Total**	
	***N***	**%**	***N***	**%**	***N***	**%**
20 –⊣ 30	1	2.33	0	0.00	1	0.93
31 –⊣ 40	1	2.33	6	10.34	7	6.54
41 –⊣ 50	6	13.95	6	10.34	12	11.21
51 –⊣ 60	8	18.60	15	25.86	23	21.50
61 –⊣ 70	17	39.53	25	43.10	42	39.25
71 –⊣ 80	8	18.60	5	8.62	13	12.15
81 –⊣ 90	2	4.65	1	1.72	3	2.80
Total	43	100	58	100	107	100

DM – diabetes mellitus, DN – diabetic nephropathy. The data are shown as frequency absolute and relative. *p* < 0.05 = level of significance.

**Table 3:** Fasting glycemia rate in patients with DN.

	Reference values	Male		Female		Total	
		*n*	%	*N*	%	*n*	%
Normal Fasting Glycemia	<110 mg/Dl	6	5.94	7	6.93	13	12.87
Altered fasting glycemia	110 mg/dL e 125 mg/dL	5	4.95	0	0.00	5	4.95
Diabetes	equal to or greater than 126 mg/dL	47	46.53	36	35.64	83	82.18
Total						101	100

**Table 4:** Clinical features of the patients with DN.

**Variables**	**Male**		**Female**		***p*-Valor**	**Total**	
	$\bar{X}$	±	$\bar{X}$	±		$\bar{X}$	±
Fasting plasma glucose (mg/dL)	192.51	81.79	214.90	96.47	0.23	202.65	93.30
Creatinine (mg/dL)	7.45	3.48	5.32	3.34	0.002*	6.54	3.62
HbA1C (%)	7.39	1.78	7.96	2.46	0.26	7.64	2.11
Pre-hemodialysis urea (mg/dL)	105.24	26.80	120.88	39.26	0.06	111.10	32.70
Post-hemodialysis urea (mg/dL)	35.70	20.28	50.46	18.07	0.07	39.40	20.50
BMI (kg/m^2^)	28.18	4.67	26.26	4.84	0.27	25.64	4.75
GFR^a^ (mL/min/1,73 m^2^)	22.33	32.14	33.91	44.94	0.15	27.34	39.12
DAP (mmHg)	77.67	9.92	76.83	10.90	0.69	77.32	10.30
SAP (mmHg)	134.24	17.94	135.52	26.26	0.73	134.92	21.77

The data are shown as averages ($\bar{X}$), standard deviation (±). *p* < 0.05 = level of significance.

**Table 5:** Blood pressure of the patients with DN.

	Pressure reference values		Blood pressure				*χ*^2^	DL	*p*		
			Male		Female					Total	
	Systolic mmHg	Diastolic mmHg	*n*	%	*n*	%				*n*	%
Normal	less than 120	less than 80	15	14.85	9	8.91	30.82	3	<0.001	24	23.76
High	120–129	less than 80	0	0	17	16.83				17	16.83
Hypertension phase 1	130–139	80–89	15	14.85	2	1.98				17	16.83
Hypertension phase 2	140 or higher	90 or higher	28	27.72	15	14.85				43	42.58
Total										101	100

The data are shown as averages ($\bar{X}$), standard deviation (±) and frequency absolute and relative. *χ*^2^: chi-square; DL: degree of freedom; *p* < 0.05 = level of significance.

**Table 6:** Distribution of patients by the mass index criteria.

Classification	Criteria	Male	%	Female	%	Total	%
Low weight	<18,5	4	6.90	1	2.33	5	4.95
Normal	≥18,5 and <25	28	48.28	20	46.51	48	47.52
Overweight	≥25 and <30	17	29.31	12	27.91	29	28.71
Obese	≥30	9	15.52	10	23.26	19	18.81
Total		58	100	43	100	101	100

The data are shown as frequency absolute and relative.

**Table 7:** Lifestyle variables across patients.

Lifestyle variable		Male	%	Female	%	*p*-Valor	OR	IC (95%)	Total	%
Smoking	Yes	9	13.43	2	4.65	0.2	3.15	0.60–31.48	11	10.00
	No	58	86.57	41	95.35				99	90.00
Alcoholism	Yes	10	17.24	5	11.63	0.57	1.58	0.44–6.39	15	14.85
	No	48	82.76	38	88.37				86	85.15
Diet	Yes	18	31.03	10	23.26	0.50	1.48	0.56–4.11	28	27.72
	No	40	68.97	33	76.74				73	72.28
Regular physical activity before DM diagnosis	Yes	37	36.21	15	34.88	0.005	3.25	1.33–8.17	52	51.49
	No	21	63.79	28	65.12				49	48.51
Regular physical activity after DM diagnosis	Yes	20	34.48	23	53.49	0.07	0.46	0.19–1.11	43	42.57
	No	38	65.52	20	46.51				58	57.43

The data are shown as averages frequency absolute and relative. OR and IC was calculated from Fisher׳s Exact Test.

**Table 8:** Influence of diet on DN patients.

**Variables**	**Without diet**		**With diet**		***p*-Valor**
	$\bar{X}$	±	$\bar{X}$	±	
Fasting plasma glucose (mg/dL)	221.83	104.68	195.77	88.71	0.28
Creatinine (mg/dL)	7.46	2.22	6.17	3.99	0.04*
HbA1C (%)	7.49	1.81	7.71	2.24	0.66
Pre-hemodialysis urea (mg/dL)	125.79	48.3	116.59	30.26	0.54
Post-hemodialysis urea (mg/dL)	49.32	20.81	54	8.49	0.67
BMI (kg/m^2^)	26.01	4.76	24.68	4.66	0.21
GFR^a^ (mL/min/1,73 m^2^)	10.23	3.47	34.29	44.56	<0.001*
DAP (mmHg)	81.43	8.48	75.74	10.55	0.006*
SAP (mmHg)	138.21	22.19	133.66	21.59	0.3582

The data are shown as averages ($\bar{X}$), standard deviation (±). *p < 0.05 = level of significance.

## 2 Experimental design, materials and methods

The data were obtained during two years (2016–2017) in 101 diabetic nephropathy (DN) patients hemodialysis treatment from the medical centers of the metropolitan region from Goiânia, GO, Brazil. The information on demographic features, lifestyle, time with type 2 Diabetes mellitus and the main exams associated with DN control were collected through questionnaires and clinical records’ analysis.

The clinical variables, the fasting plasma glucose, HbA1c (glycohemoglobin), creatinine, pre- and post-hemodialysis blood urea levels, glomerular filtration rate (GFR), systolic arterial pressure (SAP), diastolic arterial pressure (DAP), and body mass index (BMI) were obtained, too. Lastly, for the lifestyle variables, the physical activity, eating habits, alcohol consumption and smoking from all the 101 patients were characterized. The GFR was estimated by the Cockfrot-Gault formula, which considers the levels of creatinine, weight, and age. The criteria established by the American Diabetes Association [2] were used as a reference for the analyses.

These data were conducted following the ethics statement from the Helsinki Declaration and was approved by the Institutional Ethics Committee (No. 195/11 of Jun 27, 2011). All the participants signed a Free and Informed Consent Form.

Data about life, occupational history, smoking history, alcohol consumption, general health conditions, previous diseases, and other anamnesis were obtained during interviews with the patients. Only patients who had smoked for at least one year before the DM diagnostic were considered as smokers. For alcohol consumption, some individuals reported drinking only occasionally or socially.

The values for *p* < 0.05 was considered as statistically significant. All statistical analyses were conducted using RStúdio software (v.1.0.153).
