# Supplementary material for: Clinical data and risk factors for diabetic nephropathy in Brazilian central population
Source: Data Brief. 2018 Oct 27;21:1315–20. doi: 10.1016/j.dib.2018.10.115 (PMC6231041; doi:10.1016/j.dib.2018.10.115)
Supplement: Supplementary file 1 — Supplementary material. [file mmc1.doc]

# Conflict of Interest Form

All authors have participated in conception and design, or analysis and interpretation of the data; drafting the article or revising it critically for important intellectual content; and approval of the final version. This manuscript has not been submitted to, nor is under review at, another journal or other publishing venue. The authors have no affiliation with any organization with a direct or indirect financial interest in the subject matter discussed in the manuscript.

Sincerely.

Angela Adamski da Silva Reis, Ph.D.

Biological Sciences Institute (ICB), Federal University of Goiás / UFG Tel.: +55 62 3521-1431 / +55 62 9189 2070 Campus II – Esperança Avenue s/n, CEP: 74690-900, Goiânia/ GO – Brazil E-mail address: [angeladamski@gmail.com](mailto:angeladamski@gmail.com)
